# Rate of saphenous vein occlusion and side effects at 1 year follow up after 1470 nm endolaser

**DOI:** 10.1590/1677-5449.202101812

**Published:** 2023-07-21

**Authors:** Leonardo Zelotti Movio, Marco Antônio Forastieri Mansano, Marcelo Eckert Zanoni, Nancy Christiane Ferreira Silva, Marcel Pereira Rangel

**Affiliations:** 1 Universidade Cesumar - UniCesumar, Maringá, PR, Brasil.; 2 Federal de São Paulo - UNIFESP, São Paulo, SP, Brasil.; 3 Universidade Estadual de Maringá - UEM, Maringá, PR, Brasil.

**Keywords:** laser treatment, saphenous vein, varicose veins, venous insufficiency

## Abstract

**Background:**

Use of endolaser for chronic venous disease involves choosing the laser wavelength and optical fiber to use and the quantity of energy to be administered. Efficacy is assessed by the venous occlusion rate and safety is evaluated in terms of side effects.

**Objectives:**

To determine the incidence of total post-endolaser saphenous vein occlusion at 1-year follow-up. To describe side effects and their incidence and rates of reintervention or supplementary treatment during the postoperative period.

**Methods:**

A retrospective, observational cohort study with a quantitative approach, enrolling patients with saphenous vein incompetence treated with intravenous 1,470 nm laser ablation. Data were input to an MS Excel 2019 spreadsheet, calculating means and standard deviations with the software’s Power Query supplement.

**Results:**

38 patients and 104 venous segments were eligible for the study. 100% were occluded at 30 days and 99.04% were still occluded at 1 year after the procedure. Mean Linear Endovenous Energy Density administered to the internal saphenous vein was 2,040.52 W/cm/s with standard deviation of ± 1,510.06 W/cm/s and 1,168.4 W/cm/s with standard deviation of ± 665.011 W/cm/s was administered to the external saphenous vein. Pain along the saphenous path was the most common side effect, with eight cases (21.05%), followed by one case of paresthesia (2.63%).

**Conclusions:**

The total occlusion rate at 1-year follow-up suggests the technique is promising and is currently applicable in this sample. The incidence of pain and paresthesia may be caused by the high mean energy delivered in some cases. It is recommended that multicenter studies be conducted with larger and more uniform samples in terms of their Clinical-Etiological-Anatomical-Pathological classifications.

## INTRODUCTION

It is known that the clinical expression of chronic venous disease (CVD) has a wide spectrum of manifestations, varying from asymptomatic cases, with esthetic problems including telangiectasies or reticular veins, to severe symptomology, such as dermatofibrosis and ulcerations.^1^,^2^ It should be mentioned that identical manifestations may have different pathophysiological origins, varying between the several different mechanisms, such as valve incompetence, venous obstruction, and/or muscle pump dysfunction.^2^

There are few good quality longitudinal studies to confirm the incidence and prevalence of CVD in the general population and there are discrepancies between those that do exist in terms of their methodology and consequent results.^3^ However, CVD is considered one of the most common diseases of the lower limbs in the adult population, with an ever growing demand for treatments.

The largest epidemiological study in the Brazilian population is still a 1986 publication by Maffei et al. that assessed 1,755 patients at routine appointments at a University health center in Botucatu (SP), which reported a 47.6% prevalence of all types of varicose veins, with the highest rate among women who were not pregnant, at 50.9%. Cases considered moderate or severe were detected in 21.2%, even though only 5.5% of the patients had visited the health center for consultations related to varicose veins or CVD.^4^

In general, treatment for CVD will be recommended if the patient has relevant symptoms, clinical signs of chronic venous disease, and reflux in venous segments, primarily in the great and/or small saphenous veins.^5^ Depending on the recommendation, management can be conservative or with interventional surgery, which is the gold standard for treatment of varicose veins.^6^,^7^ Surgical procedures have a long history, running through a variety of methods and with a range of modifications over the years, of which saphenectomy with ligature at the saphenofemoral junction (SFJ) and saphenopopliteal junction (SPJ) have been the first choice for the great saphenous vein (GSV) and small saphenous vein (SSV), respectively, for a long time.^8^

Use of lasers for endoluminal treatment emerged after a publication by Boné (apud Hamdan).^9^ Since then, intravenous laser thermal ablation (also known as endovenous laser ablation, EVLA) began to be used for varicose veins and the classic surgical methods began to be questioned, not only because of their invasivity, but also because of the time taken for recovery, the need for hospital admission, the side effects, and the postoperative complications.^9^ Currently, according to the American Venous Forum, EVLA is strongly recommended for treatment of saphenous vein incompetence because of its safety and effectiveness and also because it requires less time for convalescence, and reduced pain and morbidity when compared to open surgery.^10^

One metric for analysis of therapeutic success of EVLA is the occlusion rate, which is one of the principal markers, primarily when analyzed with follow-up over time, while others include the number of side effects and the need for reoperation.

Taking into account the still scant number of publications specifically about EVLA using different operating techniques and considering that it is a relatively new and evolving method, especially in the Brazilian context, this research output article will present the clinical results achieved with the methodology, contributing to the attempt to perfect the treatment, achieving the greatest efficacy and least invasivity.

The primary objective of this study was to demonstrate the incidence of total venous occlusion using 1,470 nm endolaser to treat venous segments, as confirmed with Doppler ultrasonography, at 30 days and 1 year postoperative. An additional objective was to describe the side effects and their incidence during the postoperative period and report rates of reintervention or supplementary treatment.

## METHODS

This is a retrospective, observational cohort study with a quantitative approach, enrolling patients with lower limb CVD treated with EVLA at a vascular surgery service. All data were collected and analyzed retrospectively from preoperative patient records and post-laser ablation charts. The project was approved by the Ethics Committee at the Unicesumar institution, under CAAE number 15333619.9.00005539 and consolidated opinion number 4.736.805.

The present study applied the following patient eligibility criteria: having undergone EVLA of the great and small saphenous veins to treat CVD; conducted between March 2018 to October 2019; having a Clinical-Etiological-Anatomical-Pathological (CEAP) class of C2 to C5; having had Doppler ultrasonography 1 year after the procedure; and having signed a free and informed consent form (FICF).

At the clinic in question, all patients are prescribed laser thermoablation as treatment of choice and vein stripping is only indicated if the patient refuses EVLA or has a venous aneurysm. In cases in which venous dilatation was up to 12 mm from the saphenofemoral junction, treatment included ligature of the SFJ.

In a hospital surgery setting, all patients were given spinal anesthesia before undergoing EVLA with a diode endolaser at a wavelength of 1,470 nm with a 600 micra radial fiber. The procedure was started with insertion of the optical fiber from the distal point of venous insufficiency up to 0.5 cm distal of the SFJ or SPJ, under Doppler ultrasound guidance. Perivascular tumescence of the venous segment to be treated was obtained with chilled saline and, with the patient in the Trendelemburg position, irradiation with the intravenous laser was started at a cranial-caudal traction velocity of 1 mm per second. At the end of the procedure, the Linear Endovenous Energy Density (LEED) was calculated in watts per centimeter per second.

At the end of the procedure, analgesia with nonsteroidal anti-inflammatories was prescribed for 5 days and 20 to 35 mmHg elastic compression stockings for 48 hours. Patients were also encouraged to start walking immediately after hospital discharge, which was on the same day as the procedure, about 3 to 4 hours after it had been completed.

The clinical features extracted for analysis from preoperative medical records were age, sex, venous segment involved, extent of venous insufficiency, and diameter of the saphenous vein. The variables extracted from the surgical chart were power, in watts, and LEED (W/cm/s). Postoperative data obtained from the 30-day and 1-year follow-ups were: occlusion rate according to Doppler ultrasound, need for reintervention or supplementary treatment, and side effects such as skin hyperpigmentation, burning sensations, pain along the course of the vein, and paresthesia, which were analyzed as presence or absence, with no scales or grading instruments, plus deep venous thrombosis and pulmonary embolism, assessed according to echography findings combined with patients’ clinical characteristics during the postoperative period.

For the purposes of this study, the occlusion rate is defined as the percentage of lumen obliterated by EVLA after the procedure, considering 100% as being when there is no recanalization whatsoever at any point along the path of the vein. As such, recanalization is defined as any percentage of obliteration that has been reversed.

After arranging the data in an MS Excel 2019 spreadsheet, all calculations of means and standard deviations were performed using the program’s Power Query supplement.

The sample size calculation with 95% confidence interval employed the 98.1% occlusion rate described by Silva et al.^11^ as a reference and a ±3% standard error. The population is defined as infinite (finite uncountable).

For the study in question, the sample size would be approximately 80 patients who underwent the procedure on venous segments with an estimated confidence interval from 95.1% to 100% for the 1-year occlusion rate.^12^,^13^

Missing data were removed from the analysis so they would not be presented in the text or tables. No information with any relation to author bias was used.

## RESULTS

From March 2018 to October 2019, the Clinivasc vascular surgery service diagnosed 658 patients with CVD caused by saphenous vein involvement, 112 of whom had indications for surgical treatment. Forty patients were eligible for the analysis according to the predefined inclusion criteria and 72 were excluded because they did not have treatment, were treated with a different method from EVLA, had conservative treatment, or did not meet the inclusion criteria.

**Figure 1:**
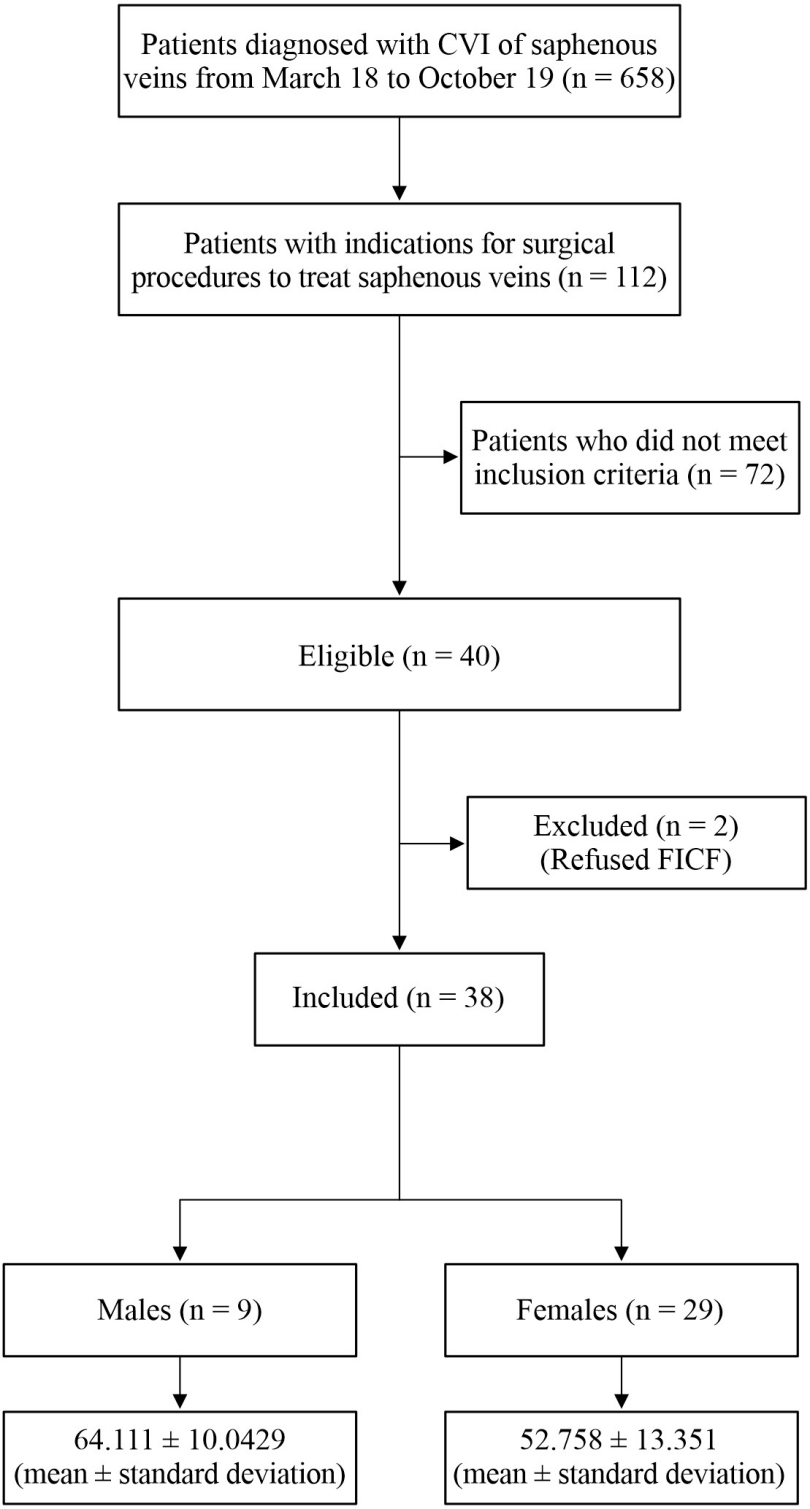
Flow diagram of the profile and selection of patients treated with endovenous laser ablation (EVLA) for the study. CVI = chronic venous insufficiency; FICF = Free and informed consent form.

A total of 38 patients were enrolled, with two excluded because they refused the FICF. One of the eligible patients was not followed-up at 1 year. Figure 1 illustrates selection, inclusion, and exclusion of patients, with their sex and mean age.

Of the 38 patients enrolled, 76 lower limbs and 104 venous segments were treated, the majority of which were GSVs, with 94 segments, 50 in left limbs and 44 in right limbs, while there were 10 SSV segments, three in left and seven in right limbs. Table 1 lists the energy density employed, calculated in W/cm/s and shown as mean LEED.

**Table 1:** Distribution of 104 vein segments treated with 1,470 nm endolaser and the energy employed.

**Venous segment treated**	**n (%)**	**Energy density**
		**(LEED)/(W/cm/s)**
Internal saphenous vein	94 (90.38)	2,040.52±1,510.06
External saphenous vein	10 (9.62)	1,168.4±665.011

LEED = linear endovenous energy density (mean ± standard deviation).

The unit of analysis for occlusion rate and LEED was the venous segment (104). For side effects, the unit was the patient (38).

As shown in Table 2, venous segment occlusion rate according to Doppler ultrasound examination at 30 postoperative days was 100%, for all 104 segments. One year after treatment, just one venous segment had undergone partial recanalization, accounting for 0.96% of the sample, and this was the only case that needed supplementary treatment, which was a sclerotherapy session. No EVLA reinterventions were performed in any of the patients.

**Table 2:** Number of venous segments totally occluded with 1,470 nm endolaser at 30 days and 1 year.

**Follow-up**	**n**	**Segments occluded**	**% of segments**
30 days	104	104	100%
1 year	104	103	99.04%

The highest incidence among the side effects analyzed at 30 days and 1 year was pain along the path of the saphenous vein after occlusion (Table 3). Regarding specific cases related to the procedure as a whole, single episodes were recorded of pain within the lumbar nerve dermatome; hematoma and burning sensation; boot erythema; persistent edema; and headaches.

**Table 3:** Side effects after thermoablation with 1,470nm endolaser in 38 patients.

**Side effect**	**n (%)**
Paresthesia	1 (2.63)
Pain along saphenous vein path	8 (21.05)
Persistent pigmentation	0 (0)
Deep vein thrombosis	0 (0)
Skin necrosis	0 (0)
Pulmonary embolism	0 (0)

## DISCUSSION

This study suggests that treatment of varicose veins with EVLA has great efficacy, considering the horizon of up to 1 year after treatment, shown by the high rates of occlusion maintained over this period and the low incidence of side effects, their benign character, and their relative facility of resolution.

The efficacy of this method consists of emitting thermal energy generated by the laser, causing irreversible damage to the wall of the vessel, primarily by denaturing collagen, which occurs from 70 to 100º C, compounded by provocation of inflammatory and fibrogenic reactions that lead to permanent occlusion of the incompetent vein.^11^,^14^ In this article, we consider the determinant factors of therapeutic success to be the radial fiber, the diode laser, the 1,470 nm wavelength, and the operating technique employed.

Our data reveal extremely elevated occlusion rates, both at 30-day follow-up (100%) and at 1 year (99.04%), with just a single venous segment exhibiting partial recanalization (0.96%). These are superior figures to the standards reported by several authors of similar studies.^7^,^11^,^15^,^16^ One similar publication, by Silva et al.,^11^ using EVLA with identical wavelengths and fibers, analyzed 180 venous segments treated, observing similar occlusion rates at 97.22% after 30 days and 98.10% at 1 year.

Galanopoulos et al.^7^ state that most studies reported occlusion rates of approximately 100% at 1 week, with this number falling over time, but remaining above 90% in many series. Moreover, the authors traced a directly proportional correlation between the quantity of energy and the occlusion rate, which is also suggested by the present study.

Although no analysis was conducted of the patients beyond 12 months, other authors believe that in the immense majority of cases, recanalization of venous segments occurs within the first 3 postoperative months, and in cases with occlusion beyond 12 months, the likelihood of future recanalization is lower when a comparison is made.^7^

In the present study, specifically with relation to LEED and occlusion rate, the results were not stratified by CEAP, which is a point that could be taken into consideration with the heterogeneous nature of the sample and as a suggestion for future studies. Nevertheless, it is relevant to mention that there is not necessarily any proportionality between the CEAP classification’s clinical item (C) and venous diameter, which is a determinant factor in the LEED calculation and thermal ablation.

The laser devices used for thermal ablation are monochromatic, i.e., they each emit a single light wavelength close to infrared, although many different wavelengths can be used (810, 940, 980, 1,064, 1,320, 1,470 and 1,980 nm). Each wavelength has a dominant tissue chromophore, i.e., the substance or tissue with the highest absorption rate, with hemoglobin predominating at bands up to 1,064 nm and then water primarily from 1,100 nm onwards.^16^,^17^

The choice of the 1,470 nm wavelength is based on the fact that this value provokes up to 40 times more absorption by the water molecules when compared with hemoglobin at the same wavelength. This factor is of interest in treatment, since using hemoglobin as the target causes a huge thrombotic phenomenon, but also a proportional effect on thrombolytic system activation, which is an important factor predisposing to recanalization and, consequently, therapeutic failure, whereas, with water, molecular excitation is predominantly in the vein wall, which is the treatment’s target site.^7^

It should be pointed out that, although the wavelength employed is focused on the water molecules in the vascular endothelium, blood cells are on average 60% water molecules and, as such, also absorb a great quantity of energy, producing coagulation, although at lower proportions than wavelengths that focus directly on hemoglobin.^17^

Therefore, in addition to the fact that its dominant chromophore is water, the 1,470 nm diode laser is also preferred because of the smaller quantity of energy needed, since devices with longer wavelengths require lower energy densities and lower power settings to achieve the therapeutic effect.^5^,^14^,^16^ In this case, use of less energy and power implies a lower likelihood of excessive heat absorption, avoiding carbonization, perforation of the wall, and postoperative pain and ecchymosis.^14^,^18^

Aktas et al.^16^ conducted a comparative study of wavelengths, observing, 7 (8.90%) recanalizations in patients treated with 980 nm and two (2.27%) in patients treated with 1,470 nm at 1 year after EVLA, out of 78 and 74 venous segments respectively.

Radial fibers were launched onto the market in 2008 and are now the most widely used type of fiber, especially for 1,470 nm diode lasers.^5^,^15^ Their superiority is due to the quartz tip, which refracts its electromagnetic beam radially in a uniform manner, reducing penetration and perforations, with fewer side effects such as pain and hematoma.^5^,^14^-^16^ Another study that compared radial and linear fibers found that the radial fiber needed less energy to achieve occlusion.^18^ Use of 600 micra fibers, which have a larger diameter and dissipate a greater energy density, enables higher final temperatures, permitting better heat distribution and conduction to the vascular tunics.^11^

The standard for describing the energy used in ablative procedures is LEED, measured in joules per centimeter by the great majority of authors. However, based on the physical definitions applied to the laser, LEED originates from the ratio of the power of the laser, measured in watts, multiplied by the velocity of fiber traction, measured in centimeters per second, so the measurement unit of LEED would be expressed as W/cm/s.^17^

In this study, laser power was calculated individually for each patient, primarily based on the measurement of the diameter of the insufficient vein, taking into consideration other determinant factors, such as the radial fiber, the velocity of reflux, and the number of tributary veins. The mean fiber traction velocity was 1 mm/s, which is the standard recommendation for segments of up to 10 mm.^14^ The mean LEED for the internal saphenous vein was 2,040.52±1,510.06 W/cm/s and 1,168.4±665.011 W/cm/s was used for the external saphenous vein.

The Trendelemburg position is used while retracting the fiber because it yields saphenous veins containing a considerably reduced quantity of intravascular blood, since large quantities would allow a high proportion of the energy to be absorbed by blood cells, reducing the energy available for the vein wall, in addition to strongly inducing the coagulation cascade, provoking recanalization.^5^,^11^ Vascular tumescence is valued for its capacity to protect perivascular tissues, acting to dissipate the heat and also to increase the luminal contact area by reducing the vein diameter.^7^,^14^

The only case of recanalization involved a patient with a C_4_ preoperative classification and factors that could possibly be considered involved in this outcome include presence of thrombophlebitis prior to treatment, which would provoke histological changes to the thickness of the venous wall because of fibrotic tissues, which could reduce the ablative effects; and the fact that the patient was 75 years old and had already been living with venous vascular disease for a long period of time and, because of this, probably had intimal and medial layers that were significantly thicker and responded less to ablation.^11^ The patient in question had persistent edema. In this case, the treatment was supplemented with a single session of sclerotherapy at the site of recanalization, after which the patient’s clinical progress was as desired, and the treatment was concluded.

Nowadays, many authors consider that the complications and postoperative side effects of EVLA are minimal, particularly when compared with vein stripping.^9^,^15^ The patients in this sample were allowed to walk on the same day as the procedure and returned sooner to their daily activities and jobs.^6^

In general, this study observed similar proportions of adverse effects to other publications, with no severe or permanent side effects.^11^,^19^,^20^ The phenomenon of postoperative pain was quite prominent in our data and in some cases, it was associated with local erythema. However, it was resolved within 30 days with nonsteroidal analgesia.

With relation to possible reasons for pain and paresthesia, no injuries were observed due to endothelial perforation by the fiber with extravascular administration of energy to adjacent tissues or nerve branches. Two pathophysiologic situations were therefore identified as possibly responsible for the symptoms. The first would be use of elevated LEED combined with a reduced fiber traction velocity which could cause tissue damage to nerve branches close to the saphenous vein because of elevated temperature. A second possibility would be failure of the perivascular tumescence to achieve sufficient distance between the nerve branches and the fiber, allowing transfer of heat and causing injury.

It is believed that the cases in which LEED was higher than average were selected cases in which the surgeon’s preoperative and intraoperative clinical analysis revealed a need to administer additional energy to achieve total obliteration of the venous segment, confirmed by the high standard deviation of energy level compared to the mean for the sample.

It is believed that, in general, it is possible that use of lower LEEDs combined with the chilled tumescence administered at the time of ablation to form a liquid halo offering thermal protection and increasing the distance to nerve branches would have attenuated patient symptomology.

The only case of paresthesia occurred after a procedure involving the left internal saphenous vein and normal sensitivity returned within 6 months. There is an up to 7% risk of nerve damage after laser ablation because of the possibility of thermal insult from the veins.^6^ The result is aggravated if tumescence with chilled saline is not administered. The same patient also had headaches after spinal anesthesia. Another patient complained of pain within the dermatome of the left lumbar nerve, also in response to the anesthesia, with remission after anticonvulsant and antiepileptic medication for 15 days.

Finally, there was a predominance of female patients in this study, which is a tendency confirmed in other publications.^21^,^22^ Explanations for this involve aspects ranging from family history, a relationship with pregnancy, and even the esthetic appearance of the lower limbs, which is also confirmed by the mean age of the female subset being 11.353 years younger than the age of the male subset, and by the larger standard deviation, at 13.351 years. Table 4 lists details of the profile of the patients studied.

**Table 4:** Patients’ demographic data (n =38).

**Sex**	
Female - n°. (%)	29 (76.32%)
Male - n°. (%)	9 (23.68%)
Age	
Male - mean. (SD)	64.1 (±10.04)
Female - mean. (SD)	52.8 (±13.35)
Distribution of saphenous vein operations	
1 x IS - n°. (%)	6 (15.78%)
2 x IS - n°. (%)	23 (60.49%)
1 x IS and 1 x ES - n°. (%)	2 (5.26%)
2 x IS and 2 x ES - n°. (%)	5 (13.15%)
1 x IS and 2 x ES - n°. (%)	2 (5.26%)

SD = standard deviation; ES = external saphenous; IS = internal saphenous.

The following should be considered as limitations of this study: it lacks follow-up beyond 1 year; does not report preoperative clinical features or diameter of the saphenous veins because of a lack of data; the sample is heterogeneous in terms of CEAP; it has no control group; the sample is relatively small; the chart template for postoperative consultations used at the service prevented insertion of additional information such as transitory pigmentation or greater detail on other side effects; the postoperative assessments were conducted by the authors; and the research was restricted to a single treatment center.

With regard to the small sample size, even though the study has revealed important findings, it should be pointed out that the ideal sample size (80 patients) would have ensured more robust results, providing better evidence on the efficacy and safety of the procedure. Moreover, the postoperative charts do not detail the specific site of adverse effects, only stating that they had occurred after the procedure.

In view of the sample described and the statistical analysis conducted, the nine patients with side effects were not considered relevant in terms of questioning the therapeutic viability of the technique, particularly since the prognosis and outcomes of the great majority of the symptoms were benign. It can also be observed that the occlusion rates at 30 days and 1 year, of 100% and 99.04% respectively, were satisfactory and similar to references from the literature mentioned above, permitting the conclusion that, in this context, the technique was promising in the sample analyzed. However, it is not possible to make recommendations based on strong evidence.

In the context of endolaser treatment for CVD, this study has made contributions in several areas: the current applicability of EVLA at 1 year post-treatment, as demonstrated and compared with similar authors; the technique employed that made it possible to achieve these results, which includes the choice of a diode laser with a wavelength tuned to water as dominant chromophore, a large diameter radial fiber, the Trendelemburg position during the procedure, and the perivascular tumescence with chilled saline; the epidemiological profile of the patients who sought treatment for CVD; possible factors that determine side effects and their respective outcomes; and description of how EVLA functions, in terms of the physical and histological mechanisms involved.

However, promising the results of this study, notwithstanding the limitations described above, it can only be considered as a starting point for further research into treatment of insufficient saphenous veins, primarily multicenter analyses and especially with respect to sample size and homogeneity, correlation with venous diameter, and patients’ CEAP, and making comparisons with other methods of treating the disease.

Since this is a retrospective cohort with the limitations that have already been covered in this material, this study can be classified as evidence level 2b.
