# Supplementary material for: Rate of saphenous vein occlusion and side effects at 1 year follow up after 1470 nm endolaser
Source: J Vasc Bras. 2023 Jul 21;22:e20210181. doi: 10.1590/1677-5449.202101812 (PMC10421577; doi:10.1590/1677-5449.202101812)
Supplement: Translation

# Translation: Taxa de oclusão em veias safenas pós-*endolaser* de 1.470 nm e efeitos colaterais no seguimento de 1 ano

**DOI:** 10.1590/1677-5449.202101811

**Authors:** Leonardo Zelotti Movio, Marco Antônio Forastieri Mansano, Marcelo Eckert Zanoni, Nancy Christiane Ferreira Silva, Marcel Pereira Rangel

**Affiliations:** 1 Universidade Cesumar - UniCesumar, Maringá, PR, Brasil.; 2 Federal de São Paulo - UNIFESP, São Paulo, SP, Brasil.; 3 Universidade Estadual de Maringá - UEM, Maringá, PR, Brasil.

**Keywords:** terapia a *laser*, veia safena, varizes, insuficiência venosa

## Abstract

**Contexto:**

O uso do *endolaser* para doença venosa crônica envolve a escolha do comprimento de onda, fibra óptica e energia dispensada. Sua eficácia é avaliada pela taxa de oclusão venosa e, a segurança, pelos efeitos colaterais.

**Objetivos:**

Demonstrar a incidência de oclusões venosas totais de veias safenas pós-*endolaser* no seguimento de 1 ano. Descrever a incidência e os efeitos colaterais e a necessidade de reintervenção ou complemento da terapêutica no pós-operatório.

**Métodos:**

Estudo observacional retrospectivo de uma coorte com abordagem quantitativa de pacientes com insuficiência das veias safenas tratados com *laser* ablação endovenosa de 1.470 nm. Dados cadastrados em planilha MS Excel 2019, com cálculos de médias e desvios padrão pelo suplemento Power Query do Software.

**Resultados:**

Foram elegíveis para o estudo 38 pacientes e 104 segmentos venosos, dos quais 100% estavam ocluídos em 30 dias e 99,04% em 1 ano pós-procedimento. O *Linear Endovenous Energy Density* médio para safena interna foi de 2.040,52 W/cm/s com desvio padrão ± 1.510,06 W/cm/s e 1.168,4 W/cm/s com desvio padrão de ± 665,011 W/cm/s para safena externa. Dor no trajeto da safena foi o principal efeito colateral, com oito casos (21,05%), seguido de parestesia, com um caso (2,63%).

**Conclusões:**

Taxa de oclusão total no seguimento de 1 ano sugerindo técnica promissora e com atual aplicabilidade na amostra. A incidência da dor e parestesia podem ser justificadas pela alta média de energia utilizada em alguns casos. Recomenda-se a realização de estudos multicêntricos, com amostras maiores e mais homogêneas em relação à classificação Clínica-Etiológica-Anatômica-Patológica.

## INTRODUÇÃO

Sabe-se que a expressão clínica da doença venosa crônica (DVC) possui largo espectro de manifestações, variando de quadros assintomáticos, com problemas estéticos que apresentam telangiectasias ou veias reticulares, até sintomatologia grave, como dermatofibrose e ulcerações^1,2^ . É necessário mencionar que manifestações idênticas podem ter origens fisiopatológicas distintas, variando entre os diversos mecanismos, como incompetência valvar, obstrução venosa e/ou disfunção da bomba muscular^2^ .

Atualmente, são poucos os estudos longitudinais de boa qualidade para confirmar números de incidência e prevalência da DVC na população geral, e existem discrepâncias quanto à metodologia e aos consequentes resultados dos trabalhos^3^ . No entanto, a DVC é considerada uma das doenças mais comuns de membros inferiores na população adulta, havendo cada vez mais procura por tratamentos.

Para a população brasileira, o maior estudo epidemiológico ainda é de Maffei et al., realizado em 1986, que avaliou 1.755 pacientes em exame de rotina no centro de saúde universitário em Botucatu (SP), demonstrando que a prevalência de veias varicosas de todos os tipos foi de 47,6%, ocorrendo principalmente em mulheres não grávidas na taxa de 50,9%. Os casos considerados moderados ou graves foram constatados em 21,2%, sendo que, da amostra analisada, somente 5,5% dos pacientes foram ao serviço de saúde para consulta relacionada às veias varicosas ou por DVC^4^ .

De forma geral, o tratamento da DVC será recomendado se o paciente possuir sintomas relevantes, sinais clínicos de doença venosa crônica e refluxo de segmentos venosos, principalmente nas veias safenas magna e/ou parva^5^ . A conduta pode ser feita, dependendo da recomendação, de forma conservadora ou de maneira cirúrgico-intervencionista, sendo, essa última, o padrão-ouro para o tratamento das veias varicosas^6,7^ . O histórico de procedimentos é de longa data, percorrendo diversos métodos e com amplas modificações, sendo a safenectomia com ligadura na junção safeno femoral (JSF) e safeno poplítea (JSP), respectivamente para as veias safenas magna (VSM) e parva (VSP), o método de escolha por longo período de tempo^8^ .

O uso do *laser* para terapia endoluminal surgiu após a publicação de Boné (apud Hamdan)^9^ . A partir de então, a ablação térmica endovenosa a *laser* (*Endovenous Laser Ablation*, EVLA) passou a ser utilizada no contexto das veias varicosas, e os métodos cirúrgicos clássicos começaram a ser questionados não só pela invasibilidade, mas também pelo tempo de recuperação, pela necessidade de internamento, pelos efeitos colaterais e pelas complicações pós-procedimento^9^ . Atualmente, de acordo com o *American Venous Forum*, a EVLA possui forte recomendação para o tratamento da incompetência de veia safena por sua segurança e efetividade, além de precisar de menos tempo para convalescência, dor e morbidade quando comparada à cirurgia aberta^10^ .

Como forma de análise do sucesso terapêutico no contexto da EVLA, tem-se a taxa de oclusão como um dos principais marcadores, principalmente quando ela é analisada em seguimento de tempo, bem como a quantidade de efeitos colaterais produzidos e a necessidade de reoperação.

Tendo em vista o número ainda reduzido de publicações específicas sobre a EVLA utilizando diferentes técnicas operatórias, por se tratar de um método relativamente novo e em evolução, especialmente no contexto brasileiro, a presente produção científica visa expor resultados clínicos obtidos mediante a utilização da metodologia, bem como contribuir em busca do aprimoramento da terapêutica com maior eficiência e menor invasibilidade.

O objetivo primário deste trabalho foi demonstrar a incidência de oclusões venosas totais utilizando o *endolaser* de 1.470 nm para o tratamento de trajetos venosos, constatando, por meio da ultrassonografia com Doppler, os períodos de 30 dias e 1 ano de pós-operatório. Adicionalmente, também foi um objetivo demonstrar quais foram os efeitos colaterais e qual sua incidência no pós-operatório, bem como a necessidade de reintervenção ou complemento da operação para a terapêutica.

## MÉTODOS

Trata-se de um estudo observacional retrospectivo de uma coorte com abordagem quantitativa de pacientes com DVC em membros inferiores tratados por EVLA por um serviço de cirurgia vascular. Todos os dados foram coletados e analisados de maneira retrospectiva através dos prontuários pré-operatórios e das fichas pós-*laser* ablação. O projeto foi aprovado pelo comitê de ética da instituição Unicesumar, com o número CAAE 15333619.9.00005539 e parecer consubstanciado 4.736.805.

O presente estudo considerou como critérios de elegibilidade dos pacientes estudados: ter sido submetido a técnica EVLA nas veias safenas parva e magna para o tratamento de DVC; possuir Clínica-Etiológica-Anatômica-Patológica (CEAP) C2 a C5; realização do procedimento entre março de 2018 e outubro de 2019; ultrassonografia com Doppler 1 ano após a realização do procedimento; aceite do Termo de Consentimento Livre e Esclarecido (TCLE).

No serviço em questão, todos os pacientes têm primariamente a indicação de termoablação por *laser* como o tratamento de escolha, sendo a fleboextração somente indicada em situações de recusa do paciente e aneurismas venosos. Nos casos de dilatações venosas distando até 12 mm da junção safeno femoral, o tratamento foi direcionado para ligadura de JSF.

Em ambiente cirúrgico hospitalar, todos os pacientes sob anestesia raquimedular foram submetidos à técnica EVLA com *endolaser* diodo de 1.470 nm de comprimento de onda com fibra radial de 600 micras. O procedimento foi iniciado com a inserção da fibra óptica no ponto distal à insuficiência venosa até 0,5 cm distal a JSF ou JSP, guiada pelo ultrassom com Doppler. Foi realizada a tumescência perivascular no segmento venoso a ser tratado com soro gelado e, com o paciente em posição de Trendelemburg, iniciou-se a irradiação do *laser* endovenoso com velocidade de tração crânio-caudal de 1 mm por segundo, sendo calculado, ao final, o *Linear Endovenous Energy Density* (LEED) em watts por centímetro por segundo.

Ao final do procedimento, foram prescritas analgesia com anti-inflamatório não hormonal por 5 dias e meia elástica de 20 a 35 mmHg por 48 horas. Ainda, foi encorajado o deambular precoce imediatamente após a alta hospitalar, ocorrida no mesmo dia do procedimento após 3 a 4 horas de sua realização.

Os aspectos clínicos analisados nos prontuários do pré-operatório foram idade, sexo, segmento venoso acometido, extensão da insuficiência e diâmetro da veia safena. Na ficha operatória, as variáveis foram a potência, calculada em watts, e o LEED (W/cm/s). Os dados no pós-operatório de 30 dias e 1 ano foram: taxa de oclusão via ultrassom com Doppler, necessidade de reintervenção ou complemento da terapêutica, além dos efeitos colaterais como: hiperpigmentação de pele, ardência, dor no trajeto e parestesia, sendo avaliados somente quanto à presença ou não, não havendo escalas ou instrumentos de graduação dos mesmos, além de trombose venosa profunda e embolia pulmonar, avaliadas com base em ecografia mencionada somada a características clínicas nos pacientes no pós-operatório.

Para o estudo, entende-se por taxa de oclusão a porcentagem do lúmen obliterado pela EVLA após o procedimento, considerando-se de 100% quando não há recanalização em nenhum ponto do trajeto venoso. Dessa forma, fica também definida a recanalização como qualquer percentual em que a obliteração tenha se desfeito.

Após a estruturação dos dados cadastrados em planilha MS Excel 2019, todos os cálculos relativos às médias e aos desvios padrão foram realizados a partir do suplemento Power Query, no mesmo programa.

Para o cálculo do tamanho da amostra com intervalo de confiança de 95%, considerou-se como referência a taxa de oclusão de 98,1% descrita por Silva et al.^11^ , e ±3% de erro padrão. A população caracterizou-se como infinita (finita não enumerável).

Para o estudo em questão, o tamanho da amostra seria de aproximadamente 80 pacientes submetidos ao procedimento nos segmentos venosos com intervalo de confiança estimado entre 95,1% e 100% para a taxa de oclusão em 1 ano^12-13^ .

Dados faltantes foram retirados de análise e descontinuados de possível apresentação textual ou em tabelas. Não houve utilização de informação com relação de viés aos autores.

## RESULTADOS

Do mês de março de 2018 até outubro de 2019, o serviço de cirurgia vascular Clinivasc diagnosticou 658 pacientes com DVC devido a acometimento de veias safenas, dos quais 112 possuíam indicação de tratamento cirúrgico. Quarenta pacientes foram elegíveis para análise segundo os critérios de inclusão pré-determinados, e 72 foram desconsiderados devido a não realização/desistência do tratamento, realização cirúrgica via método não EVLA, tratamentos conservadores e por não se enquadrarem nos critérios de inclusão.

Foram incluídos 38 pacientes, havendo duas exclusões por recusa do TCLE. Não houve seguimento para além de 1 ano dos pacientes elegíveis. A Figura 1 representa a seleção de pacientes incluídos e excluídos, além dos sexos e suas respectivas médias de idade.

**Figura 1:** Fluxograma do perfil e da seleção dos pacientes tratados com a ablação térmica endovenosa a *laser* (EVLA) submetidos ao estudo. IVC = insuficiência venosa crônica; TCLE = Termo de consentimento livre e esclarecido.

Entre os 38 pacientes considerados, foram tratados 76 membros inferiores e 104 segmentos venosos, dos quais a VSM foi maioria com 94 trajetos, sendo 50 em membro esquerdo e 44 em membro direito, enquanto para a VSP, foram 10 segmentos, três em membro esquerdo e sete em direito. Quanto à densidade de energia utilizada, o cálculo foi feito em W/cm/s demonstrado em LEED médio na Tabela 1.

**Tabela 1:** Distribuição dos 104 segmentos tratados com *endolaser* de 1.470 nm e energia utilizada.

**Segmento venoso tratado**	**n (%)**	**Densidade de energia**
		**(LEED)/(W/cm/s)**
Veia safena interna	94 (90,38)	2.040,52±1.510,06
Veia safena externa	10 (9,62)	1.168,4±665,011

*LEED =* linear intravenous energy density *(média ± desvio padrão).*

A unidade de análise para a taxa de oclusão e LEED foram os segmentos venosos (104). Para os efeitos colaterais, foram os pacientes (38).

Conforme a Tabela 2, ao exame de ultrassom Doppler, a taxa de oclusão dos segmentos venosos em 30 dias de pós-operatório foi de 100%, totalizando os 104 segmentos. Após 1 ano de tratamento, houve somente um trajeto venoso com recanalização parcial, representando 0,96% da amostra, sendo esse o único caso de necessidade de complemento de terapêutica, em que foi realizada sessão de escleroterapia. Não houve reintervenções via EVLA para nenhum dos pacientes.

**Tabela 2:** Número de segmentos venosos totalmente ocluídos com *endolaser* de 1.470 nm nos períodos de 30 dias e 1 ano.

**Tempo de seguimento**	**n**	**Segmentos ocluídos**	**% de segmentos**
30 dias	104	104	100%
1 ano	104	103	99,04%

Entre os efeitos colaterais analisados no período de 30 dias a 1 ano, o de maior incidência foi a dor no trajeto da veia safena pós-oclusão (Tabela 3). Em relação aos casos específicos relacionados ao procedimento como um todo, foram registrados episódios unitários de: dor em dermátomo de nervo lombar; hematoma e ardência; eritema bota; edema persistente; cefaleia.

**Tabela 3:** Efeitos colaterais após termoablação com *endolaser* de 1.470nm em 38 pacientes.

**Efeito colateral**	**n (%)**
Parestesia	1 (2,63)
Dor no trajeto da veia safena	8 (21,05)
Pigmentação persistente	0 (0)
Trombose venosa profunda	0 (0)
Necrose de pele	0 (0)
Embolia pulmonar	0 (0)

## DISCUSSÃO

O presente estudo sugere grande eficácia do tratamento de veias varicosas pela EVLA, considerando-se o prazo de até 1 ano pós-terapêutica, fato corroborado pelas altas taxas de oclusão mantidas nesse período, pela baixa incidência de efeitos colaterais, por seu caráter benigno, além de relativa facilidade de resolução.

A eficiência do método em questão consiste em, por meio da emissão de energia térmica gerada pelo *laser*, causar dano irreversível à parede do vaso, seja primariamente pela desnaturação de colágeno, que ocorre entre 70 e 100º C, somada à evocação de reações inflamatórias e fibrogênicas, que culminam na oclusão permanente da veia incompetente^11,14^ . No artigo, consideramos como fatores determinantes para o sucesso terapêutico: a fibra radial, o *laser* diodo, o comprimento de onda de 1.470 nm e a técnica operatória utilizada.

Nossos dados revelaram taxas de oclusão extremamente elevadas, tanto para o seguimento de 30 dias (100%) quanto ao 1º ano pós-operatório (99,04%), com somente um trajeto venoso apresentando recanalização parcial (0,96%), números inclusive acima dos padrões de diversos autores com estudos similares^7,11,15,16^ . Em publicação semelhante de Silva et al.^11^ , que utilizou EVLA com comprimento de onda e fibra idênticos, foram constatadas, em 180 segmentos venosos tratados, taxas de oclusão semelhantes de 97,22% em 30 dias e de 98,10% em 1 ano dos vasos avaliados.

Galanopoulos et al.^7^ afirmaram que a maior parte dos estudos já indicava taxa de oclusão de aproximadamente 100% em uma semana, com esse número caindo ao longo do tempo, porém se mantendo acima de 90% em diversas séries. Além disso, os autores pontuaram a correlação da proporcionalidade direta da quantidade de energia e taxa de oclusão, fato também sugerido pelo presente estudo.

Embora não tenha sido feita a análise dos pacientes após 12 meses, outros autores acreditam que a recanalização de segmentos venosos ocorre, na imensa maioria dos casos, dentro dos primeiros 3 meses de pós-operatório, e que, em casos de oclusão acima de 12 meses, a chance de recanalização futura é mais improvável quando feita a comparação^7^ .

No presente estudo, especificamente quanto ao LEED e à taxa de oclusão, não houve estratificação dos resultados por CEAP, ponto a ser levado em consideração pela heterogeneidade da amostra e como sugestão para futuros estudos. Ainda assim, é cabível mencionar que não há proporcionalidade obrigatória entre a classificação em seu item clínico (C) e o diâmetro venoso, fator esse que é determinante para o cálculo do LEED e da ablação térmica.

Os dispositivos a *laser* utilizados na termoablação possuem caráter monocromático, ou seja, emitem luz única e próxima ao infravermelho, além de possuírem diversos comprimentos de onda utilizáveis (810, 940, 980, 1.064, 1.320, 1.470 e 1.980 nm). Cada comprimento de onda detém um cromóforo tecidual dominante, ou seja, substância ou tecido com maior taxa de absorção, havendo prevalência para hemoglobina, nas faixas até 1.064 nm, e para água, principalmente a partir de 1.100 nm^16,17^ .

A escolha do comprimento de onda de 1.470 nm se baseia no fato de esse valor promover até 40 vezes mais absorção pelas moléculas de água quando comparado com a hemoglobina na mesma faixa. Esse fator se mostra interessante para a terapêutica, já que a utilização da hemoglobina como alvo causa enorme fenômeno trombótico, mas proporcional efeito ativador do sistema trombolítico, sendo um fator predisponente importante para recanalização e, consequentemente, falha terapêutica, enquanto, para a água, a excitação molecular tem predomínio na parede venosa, local-alvo da terapia^7^ .

É necessário esclarecer que, embora o comprimento de onda utilizado tenha foco nas moléculas de água do endotélio vascular, as células sanguíneas são compostas, em média, por 60% das mesmas moléculas e, portanto, também absorvem grande quantidade de energia, produzindo coagulação, apesar de em menores proporções quando comparadas com comprimentos de onda que focam diretamente a hemoglobina^17^ .

Desse modo, em adição ao aspecto do cromóforo dominante água, a utilização do *laser* diodo de 1.470 nm tem sua preferência devido à menor quantidade de energia necessária a ser dispendida, isso porque os dispositivos com maior comprimento de onda necessitam de menores densidades de energia juntamente com menores potências a serem operadas para a obtenção da terapêutica^5,14,16^ . O uso de menor energia e potência, nesse caso, proporciona menores chances de absorção excessiva de calor, impedindo carbonização, perfuração de parede, dor e equimose pós-operatória^14,18^ .

Em pesquisa comparativa entre os comprimentos de onda, Aktas et al.^16^ obtiveram, em 1 ano pós-EVLA 7 (8,90%), recanalizações com 980 nm e dois (2,27%) com 1.470 nm de um total de 78 e 74 trajetos venosos respectivamente.

A fibra radial foi introduzida no mercado em 2008 e, atualmente, é a fibra mais utilizada, especialmente para o *laser* diodo de 1.470 nm^5,15^ . Seu uso tem sido atrelado à sua ponteira de quartzo, que permite refletir seu feixe eletromagnético em direção radial e de forma homogênea com menor penetração e perfurações, reduzindo efeitos colaterais como dor e hematomas^5,14-16^ . Em outro estudo que faz a comparação com a fibra linear, foi constatado que a fibra radial demanda menos energia para a obtenção da oclusão^18^ . O uso da fibra de 600 micras, devido a seu maior diâmetro e sua maior densidade energética dissipada, possibilita atingir temperaturas finais mais elevadas, permitindo melhor distribuição e condução de calor até as túnicas vasculares^11^ .

A descrição da energia para os procedimentos ablativos utiliza o LEED como padrão, mensurada pela grande maioria dos autores na unidade de joules por centímetro. No entanto, pelas definições físicas aplicadas ao *laser*, o LEED se origina da razão da potência do *laser*, medida em watts, multiplicada pela velocidade de tração da fibra, dimensionada em centímetros por segundo, logo, a unidade de medida em LEED seria expressa em W/cm/s^17^ .

Neste estudo, o cálculo da potência do *laser* foi individualizado para cada paciente, principalmente com base na medida do diâmetro da veia insuficiente, levando em consideração outros fatores delineadores, como a fibra radial, a velocidade de refluxo e a quantidade veias tributárias. A velocidade de tração da fibra obteve uma média de 1 mm/s, padrão recomendado para segmentos de até 10 mm^14^ . Como LEED para veia safena interna, obtivemos a média de 2.040,52±1.510,06 W/cm/s e, para veia safena externa, 1.168,4±665,011 W/cm/s.

A opção pela posição de Trendelemburg durante a retração da fibra se dá por essa propiciar veias safenas com redução importante na quantidade de sangue intravascular, já que a presença desse permite a alta absorção de energia pelas células sanguíneas, diminuindo a disponibilidade para parede venosa, além de induzir fortemente a cascata de coagulação, promovendo a recanalização^5,11^ . A realização de tumescência vascular é valorizada pela capacidade de proteger os tecidos perivasculares, agindo como dissipador de calor, e ainda causar aumento da área de contato luminal pela diminuição do diâmetro venoso^7,14^ .

O único caso de recanalização envolveu um paciente com classificação C_4_ no pré-operatório, sendo considerados como possíveis fatores para esse desfecho: presença de tromboflebite anterior ao tratamento, a qual provoca alteração histológica com espessamento da parede venosa por um tecido fibrótico, podendo reduzir os efeitos ablativos; e o paciente possuía 75 anos de idade e já havia convivido com doença vascular venosa por longo período de tempo e, por isso, provavelmente possuía camadas íntima e média significativamente mais espessas com menor resposta à ablação^11^ . O paciente em questão apresentou edema persistente. Para o caso, foi realizado complemento da terapêutica por meio de sessão única de escleroterapia no local, com o paciente evoluindo clinicamente de forma desejada e concluindo o tratamento.

Atualmente, as complicações e os efeitos colaterais pós-operatórias da EVLA já são considerados mínimos por diversos autores, principalmente quando comparados com a fleboextração^9,15^ . Nessa amostra, pacientes foram liberados para deambular no mesmo dia do procedimento, além de retornarem mais rapidamente às atividades diárias e à ocupação laboral^6^ .

De forma geral, a pesquisa obteve proporções semelhantes a outras quanto aos efeitos adversos, inclusive não havendo efeitos colaterais graves e permanentes^11,19,20^ . Especialmente em nossos dados, o fenômeno da dor pós-operatória teve certa relevância, estando, em alguns casos, associada a eritema local; no entanto, ela se resolveu dentro do período de 30 dias com o uso da analgesia não esteroidal.

Em relação às possíveis justificativas para a dor e parestesia, foi verificada a inexistência de lesão por perfuração endotelial pela fibra com disparo extravascular em tecidos adjacentes e ramos nervosos. Foram, no entanto, elencadas duas situações fisiopatológicas, possivelmente determinando os sintomas. A primeira foi por conta do uso de LEEDs elevados associados à diminuição da velocidade de tração da fibra que, ao aumentar a temperatura e sua difusão, possivelmente causou lesão tecidual e de ramos neurais próximos à veia safena. Em segundo lugar, possivelmente houve falha no afastamento com tumescência perivascular de ramos nervosos, determinando passagem térmica e lesão.

Quanto às ocasiões em que o LEED ficou acima da média, entende-se que esses foram casos seletos, em que, através da análise clínica pré e intraoperatória do cirurgião, foi necessária maior dispersão energética para a obtenção de obliteração completa do segmento venoso, comprovada pelo alto desvio padrão de energia em relação à média dos casos.

Acredita-se, de maneira geral, que a utilização de menores LEEDs associados à tumescência gelada realizada no momento da ablação para a formação de halo hídrico de proteção térmica e afastamento de ramos nervosos possivelmente reduza a sintomatologia apresentada pelos pacientes.

O único caso de parestesia ocorreu após procedimento na veia safena interna esquerda, retornando à sensibilidade normal antes de 6 meses. A lesão nervosa possui risco de até 7% após *laser* ablação, devido ao possível insulto térmico nas veias^6^ . O quadro é agravado caso não seja realizada a tumescência com soro gelado. Foi observada, no mesmo caso, presença de cefaleia após anestesia raquimedular. Outro paciente, também por conta da anestesia, referiu dor no dermátomo de nervo lombar à esquerda, com remissão após medicação anticonvulsivante e antiepilética por 15 dias.

Por fim, neste estudo, foi constatada a predominância de pacientes do sexo feminino, uma tendência que se confirma em outras publicações^21,22^ . A explicação envolve desde questões de histórico familiar, relação com gravidez e até pelo aspecto estético dos membros inferiores, confirmada, inclusive, pela média feminina de idade 11,353 anos inferior quando comparada ao sexo masculino e pelo maior desvio padrão, de 13,351 anos. A Tabela 4 expõe o perfil dos pacientes estudados.

**Tabela 4:** Dados demográficos dos pacientes (n =38).

**Gênero**	
Feminino - n°. (%)	29 (76,32%)
Masculino - n°. (%)	9 (23,68%)
Idade	
Masculino - média. (DP)	64,1 (±10,04)
Feminino - média. (DP)	52,8 (±13,35)
Distribuição de operações em veias safenas	
1 x SI - n°. (%)	6 (15,78%)
2 x SI - n°. (%)	23 (60,49%)
1 x SI e 1 x SE - n°. (%)	2 (5,26%)
2 x SI e 2 x SE - n°. (%)	5 (13,15%)
1 x SI e 2 x SE - n°. (%)	2 (5,26%)

DP = desvio padrão; SE = safena externa; SI = safena interna.

Devem-se considerar como limitações para este estudo: falta de seguimento além de 1 ano; não relacionar os aspectos clínicos e o diâmetro das veias safenas no pré-operatório por falta de dados; amostra heterogênea quanto ao CEAP; não possuir grupo-controle; amostra relativamente pequena; modelo de fichas para o pós-operatório utilizadas pelo serviço, impedindo a adição de outras informações, como pigmentação transitória e maior discriminação de outros efeitos colaterais; avaliação pós-operatória feita pelos autores; e a pesquisa ter sido realizada somente em um centro de tratamento.

Considerando-se o reduzido tamanho amostral, ainda que revelador de importantes achados neste trabalho, deve-se frisar que uma amostra ideal (com 80 pacientes) asseguraria resultados mais robustos, permitindo, com melhores evidências, ponderar acerca da eficácia e segurança do procedimento. Ademais, as fichas para o pós-operatório não possuíam descriminação quanto ao local específico dos efeitos adversos, somente apontavam que eles ocorreram após o procedimento.

Em vista à casuística apresentada e à análise estatística realizada, não foi constatada relevância nos nove pacientes com efeitos colaterais a ponto de questionar a viabilidade terapêutica da técnica, além de ser verificado o bom prognóstico e a evolução benigna da grande maioria dos sintomas. Também foi possível observar que as taxas de oclusão no seguimento de 30 dias e 1 ano de pós-operatório, respectivamente de 100% e 99,04%, foram satisfatórias e similares a literaturas supracitadas, permitindo apontar que, nesse contexto, a técnica foi promissora na amostra. Contudo, não houve a viabilidade de realizar recomendações com grande evidência.

No contexto da terapia com *endolaser* para a DVC, este estudo acrescenta em diversas vertentes: a atual aplicabilidade da EVLA no 1° ano pós-tratamento como demonstrado e comparado com autores semelhantes; a técnica utilizada que possivelmente proporcionou tais resultados, que inclui *laser* diodo com comprimento de onda para cromóforo dominante água, fibra radial de grande diâmetro, posição de Trendelemburg durante procedimento e tumescência perivascular com soro gelado; o perfil epidemiológico dos pacientes que procuraram por tratamento para DVC; possíveis fatores que determinaram os efeitos colaterais e seus respectivos desfechos; e descrição do funcionamento da EVLA quanto a seus mecanismos físico-histológicos.

Por mais promissores que os resultados deste estudo tenham sido, ainda que com as limitações já descritas, ele pode e deve ser apenas considerado como ponto de partida para a realização de novas pesquisas no contexto da terapêutica de veias safenas insuficientes, principalmente em análise multicêntrica e especialmente em tamanho e homogeneidade de amostra, correlação com diâmetro venoso e CEAP dos pacientes, realizando comparações com outas modalidades terapêuticas para a doença.

Por se tratar de coorte retrospectiva com as limitações já apresentadas neste material, o estudo pode ser enquadrado no nível de evidência 2b.
